# Artifactual hypoglycemia caused by Raynaud’s phenomenon: A case report with literature review

**DOI:** 10.3389/fendo.2025.1695633

**Published:** 2025-11-24

**Authors:** Hidenori Nishioka, Takeshi Yamamotoya, Yuki Itoda, Chikako Ichikawa, Mai Nishiyama, Masahiro Takubo, Akiko Nagasawa, Minami Kosuda, Fujiko Egashira, Midori Fujishiro, Kentaro Watanabe, Hisamitsu Ishihara

**Affiliations:** 1 Division of Diabetes and Metabolic Diseases, Nihon University School of Medicine, Tokyo, Japan; 2 Department of Diabetes and Metabolism, Nihon University School of Medicine Itabashi Hospital, Tokyo, Japan

**Keywords:** artifactual hypoglycemia, pseudo-hypoglycemia, Raynaud’s phenomenon, finger-stick glucose monitoring, false-low glucose value

## Abstract

Finger-stick glucose monitoring is commonly used in the clinical management of diabetes as a tool to obtain a reliable estimate of venous glucose levels. However, it should be noted that discrepancies can arise in certain situations between the finger-stick glucose value and venous blood glucose concentration. We present herein the case of a 76-year-old woman with dermatomyositis presenting with artifactual hypoglycemia, in which finger-stick glucose monitoring exhibited false-low values due to Raynaud’s phenomenon. Despite the low glucose level (<54 mg/dL) on finger-stick glucose monitoring, she was asymptomatic, and occasional laboratory blood tests failed to detect apparent hypoglycemia. We suspected artifactual hypoglycemia to be caused by Raynaud’s phenomenon, and consistently, switching the blood sampling site from the finger to the earlobe ameliorated the discrepancy against the actual venous glucose levels. Given the prevalence of steroid-induced diabetes in patients with Raynaud’s phenomenon, clinicians should be aware that finger-stick glucose monitoring can present false-low values due to Raynaud’s phenomenon, thus avoiding unnecessary investigations searching for the cause of “hypoglycemia,” or conversely, preventing underestimation of the actual hyperglycemia.

## 1 Introduction

Hypoglycemia, which is generally defined as a blood glucose concentration of <70 mg/dL (<3.9 mmol/L) (1), typically occurs in patients with diabetes using glucose-lowering drugs or insulin. Hypoglycemia can also occur in people without diabetes, along with certain diseases and conditions, such as insulinoma, adrenal insufficiency, liver or kidney dysfunction, and postprandial hypersecretion of insulin, a phenomenon referred to as reactive hypoglycemia.

As suggested by the result of the Action to Control Cardiovascular Risk in Diabetes (ACCORD) trial (2), in which increased mortality as well as increased hypoglycemic events were observed in the intensive-therapy group, hypoglycemia can pose serious threats to life by increasing the risk of cardiovascular events. The potential mechanisms underlying hypoglycemia-induced cardiovascular events include hemodynamic changes and cardiac arrhythmias driven by the activation of the sympathoadrenal system as well as prothrombotic and pro-inflammatory responses caused by hypoglycemia (3, 4). Therefore, avoiding hypoglycemia by accurately monitoring blood glucose levels is crucial for managing patients with diabetes.

In clinical settings, finger-stick glucose monitoring is commonly performed to grasp the patient’s venous blood glucose levels, providing useful information to clinicians for adjusting the doses of insulin or oral antidiabetic agents. Self-monitoring blood glucose meters are standardized in accordance with ISO 15197, which requires at least 95% of the results to be within 15 mg/dL of actual glucose concentrations of the reference for glucose concentrations below 100 mg/dL and within 15% for concentrations equal to or above 100 mg/dL (5). Therefore, capillary glucose levels obtained from finger-stick glucose monitoring are generally considered a reliable estimate of venous blood glucose levels (6); however, it should be kept in mind that a considerable discrepancy between them can be observed in certain conditions.

Herein, we present a case of artifactual hypoglycemia observed in a patient with Raynaud’s phenomenon, in which finger-stick glucose measurement presented significantly lower glucose levels than venous blood glucose levels.

## 2 Case report

A 76-year-old woman visited the emergency department complaining of dyspnea that had lasted for a week. With ST elevation in V2–4 on electrocardiogram and global left ventricular dysfunction on echocardiogram, ischemic heart disease was initially suspected, and coronary angiography was performed. Since there was no apparent stenosis in the coronary arteries, she was diagnosed with acute decompensated heart failure due to Takotsubo cardiomyopathy (7) and was hospitalized in the coronary care unit (CCU).

She developed dermatomyositis at the age of 69 and had been receiving glucocorticoid therapy. During the course of treatment, her blood glucose levels became elevated, and she was diagnosed with steroid-induced diabetes. With the prednisolone intake of 5 mg daily, however, she maintained fair glycemic control (i.e., HbA1c < 7.0%); thus, she was not taking any hypoglycemic agents at the time of admission.

While in the CCU, her blood glucose levels were monitored periodically using arterial blood gas analyses, which revealed no apparent hypoglycemia (**Table 1A**, arterial blood gas analyzer). However, after she was transferred to a general ward on day 9, low blood glucose values were detected on regular finger-stick glucose monitoring (**Table 1A**, finger-prick blood, portable blood glucose meter), occasionally to the extent that fulfills the criteria for level 2 hypoglycemia (<54 mg/dL) (1); therefore, she was referred to our department.

Table 1Summary of the results of glucose measurements.

**A:** 

Time	Day															
	1	2	3	4	5	6	7	8	9	10	11	12	13	14	15	16
Before breakfast		136	156	130	214	138	95	152	98	85	91	79	84	72	100	69
Before lunch		164	179	176	206	177	130	133	57	71	71	71	39	63	95	50
Before dinner	326	148	137	189	149	139	116	120	138	109	83	74	119	99	106	117
Before bedtime	183	156	138	213	164	157	116	120	119	140	110	64	86	104	82	99
	**Arterial blood gas analyzer**								**Finger-prick blood, Portable blood glucose meter**							
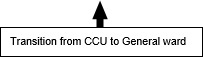																
Time	Day															
	17	18	19	20	21	22	23	24	25	26	27	28	29	30	31	32
Before breakfast	71	67	89	91	34	79	96	61	73	68	82	76	104	81	69	66
Before lunch	99	113	N/A	21	120	98	84	77	61	87	67	83	84	163	123	100
Before dinner	126	115	104	81	124	126	93	124	99	107	139	107	128	127	141	184
Before bedtime	117	123	123	117	125	103	98	133	96	97	108	103	158	106	120	103
	**Finger-prick blood, Portable blood glucose meter**				**Earlobe-prick blood, Portable blood glucose meter**											
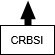

**B:** 

Sample	Glucose measurement	Day	
		21	30
Finger-prick blood	Portable blood glucose meter	34	
Earlobe-prick blood	Portable blood glucose meter		81
Venipuncture blood	Portable blood glucose meter	129	71
Venipuncture serum	Central clinical laboratory	126	90

(A) Trends of routinely measured glucose levels. Her glucose levels were monitored by arterial blood gas analyses in the CCU (days 1–9) and then by finger-prick glucose measurements after being transferred to a general ward (days 9–21). Since finger-prick measurements presented false-low values due to Raynaud’s phenomenon, we switched to earlobe-prick glucose measurements from day 21. Blue highlights indicate level 1 hypoglycemia, and yellow highlights indicate level 2 (37). The glucose levels are listed in mg/dL. CCU, coronary care unit; CRBSI, catheter-related bloodstream infection; N/A, not available.

(B) Comparison of glucose values obtained using a portable blood glucose meter (finger-prick, earlobe-prick, or venous blood) and actual venous glucose values measured in the central clinical laboratory. The glucose levels are listed in mg/dL.

She was alert and asymptomatic despite the apparent low blood glucose level in finger-stick glucose monitoring. Laboratory tests indicated no apparent endogenous hypersecretion of insulin [serum immunoreactive insulin (IRI) 2 μU/mL, C-peptide immunoreactivity (CPR) 1.37 ng/mL, corresponding to the plasma glucose level of 86 mg/dL], negative insulin autoantibody, and no apparent adrenal insufficiency (morning serum adrenocorticotropic hormone 17.2 pg/mL, cortisol 10 μg/dL). Insulinoma was unlikely, also from the result of a contrast-enhanced abdominal CT scan performed on day 19. In addition, despite the frequent low blood glucose levels observed in finger-stick glucose testing, occasional laboratory blood tests failed to detect apparent hypoglycemia.

**Figure 1:**
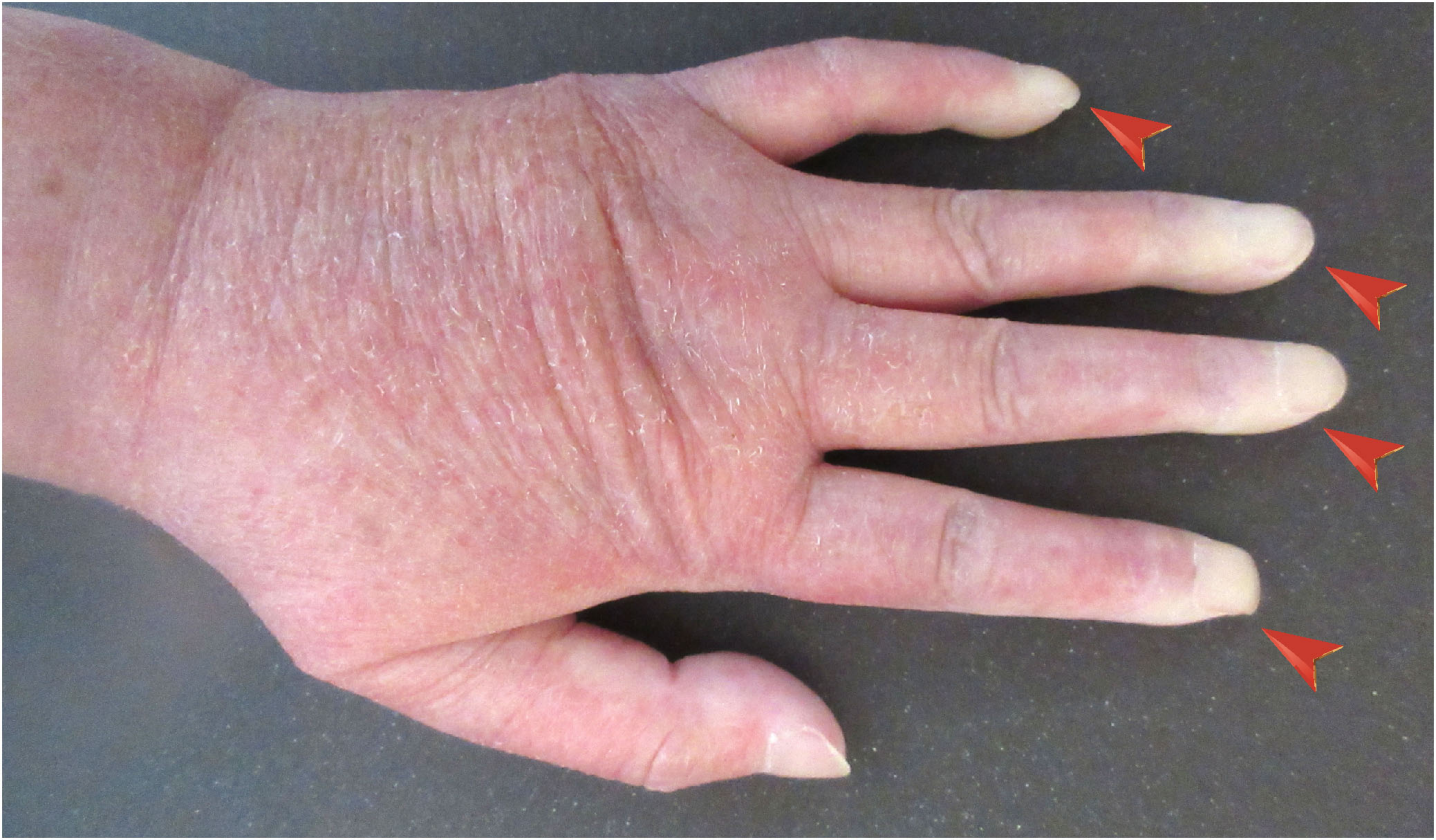
Picture of a hand from a 76-year-old woman who presented with artifactual hypoglycemia in finger-stick glucose measurements. She was diagnosed with dermatomyositis, and her fingers were pale due to Raynaud’s phenomenon (red arrowheads).

Since her “hypoglycemia” was only evident in finger-stick glucose monitoring, we hypothesized that finger-stick glucose monitoring might exhibit falsely low values for some reason(s). Therefore, we measured and compared a) finger-prick, b) earlobe-prick, and c) venous blood (obtained by venipuncture) glucose levels measured using a portable blood glucose meter, as well as d) venous plasma glucose levels measured in a central clinical laboratory. As shown in **Table 1B**, only the finger-prick blood glucose level measured using a portable blood glucose meter presented a lower value. A close physical examination revealed a pallor color change in her digits, indicating Raynaud’s phenomenon (**Figure 1**). We concluded that her low blood glucose levels in finger-stick glucose monitoring were “artifactual hypoglycemia” caused by Raynaud’s phenomenon (8).

We switched the blood sampling site for glucose monitoring from the finger to the earlobe, and extremely low glucose values (<54 mg/dL), as often detected in finger-stick testing, were not observed thereafter (**Table 1A**, earlobe-prick blood, portable blood glucose meter). The patient recovered from heart failure, and she was discharged on day 96.

## 3 Discussion

We experienced a case of artifactual hypoglycemia in which finger-stick glucose monitoring exhibited false-low values owing to Raynaud’s phenomenon. In some previous literature, “pseudo-hypoglycemia” had been used to describe the same conditions as artifactual hypoglycemia (9–12). However, since the American Diabetes Association and the Endocrine Society defined “pseudo-hypoglycemia” as an event with typical symptoms of hypoglycemia without definite hypoglycemia (≤70 mg/dL) (13), the term “artifactual hypoglycemia” was proposed by Tarasova et al. (8) to describe a discrepancy between various laboratory measurements and actual blood glucose levels, regardless of the presence or absence of symptoms. Artifactual hypoglycemia is classified into two groups: false-low capillary glucose (*in vivo*) and false-low plasma glucose (*in vitro*) (8). The former is caused by decreased capillary flow, as observed in Raynaud’s phenomenon, critically ill patients with shock, and those with peripheral vascular diseases. The latter can be caused by increased glycolysis *in vitro*, which indicates glucose consumption by blood cells after obtaining blood samples, as observed in polycythemia vera and leukemia (8).

Raynaud’s phenomenon is characterized by episodic vasospasm of the fingers and toes, typically precipitated by exposure to cold temperatures. Primary Raynaud’s phenomenon refers to cases with no underlying illness, whereas secondary Raynaud’s phenomenon is caused in association with other disorders or conditions, such as rheumatological diseases, mechanical injury (such as vibration), and abnormal blood elements (such as cryoglobulins and cold agglutinins) (14). Secondary Raynaud’s phenomenon is reportedly observed in more than 90% of patients with systemic sclerosis (SSc), 10%–45% with systemic lupus erythematosus (SLE), 33% with Sjögren’s syndrome, and 20% with dermatomyositis or polymyositis (14, 15). The artifactual hypoglycemia observed in Raynaud’s phenomenon is explained by decreased capillary blood flow and the resultant longer transit time, which leads to increased glucose extraction by peripheral tissues, rendering glucose values by finger-stick measurements significantly lower than venous glucose concentrations (8, 16).

As of 23 October 2025, a PubMed search for “Artifactual hypoglycemia and Raynaud” or “Pseudohypoglycemia and Raynaud” identified 13 articles (8–12, 17–24) discussing 14 cases of artifactual hypoglycemia induced by Raynaud’s phenomenon (**Table 2**). In most cases, patients were asymptomatic, or had symptoms suggestive of hypoglycemia but proven to be inconsistent with actual glycemic levels (10, 11). Underlying illnesses that caused Raynaud’s phenomenon were available in at least 11 cases, suggesting that most of the cases were categorized as secondary, not primary, Raynaud’s phenomenon. This is not surprising considering the higher frequency of steroid-induced diabetes and the resultant more opportunities for finger-stick glucose measurements in patients with secondary Raynaud’s phenomenon than in those with primary Raynaud’s phenomenon. However, endothelial damage, which is one of the characteristics of secondary Raynaud’s phenomenon (14), might also contribute to the higher frequency of artifactual hypoglycemia by exacerbating vasospasm and capillary blood flow, via several mechanisms including the proliferation and contraction of smooth muscle cells, increased procoagulant activity and decreased fibrinolysis, and local inflammatory processes (14). Of note, eight cases involved concomitant potentially hypovolemic or hypotensive conditions that may exacerbate peripheral hypoperfusion (8, 11, 18, 19, 21, 22, 24). In our case, the patient not only experienced acute heart failure but also developed a catheter-related bloodstream infection on day 19, both of which may have contributed to the pathogenesis of the artifactual hypoglycemia. Consistently, Atkin SH et al. reported that 32% of hypotensive patients were falsely diagnosed with hypoglycemia using finger-prick glucose measurement (25).

**Table 2:** Features of artifactual hypoglycemia induced by Raynaud’s phenomenon.

Reference	Age (years)	Sex	Underlying illness	Conditions that potentially cause peripheral hypoperfusion	Symptoms suggestive of hypoglycemia	Representative simultaneous glucose test results		Alternative glucose testing
						Finger-prick portable glucose meter	Venipuncture central laboratory	
Rushakoff RJ, et al. (2001) (11)	44	F		Gastroenteritis	Light-headedness, fatigue, sweating	42	86	
El Khoury M, et al. (2008) (10)	27	F	MCTD		Dyspnea, anxiety, tachycardia, tachypnea, sweating, tremors	42	98	Earlobe
Tarasova VD, et al. (2014) (8)	75	F		Post-esophagectomy	None			
Radosevich MA, et al. (2015) (12)	68	F	SSc		Fatigue, drowsiness			Venous and arterial blood
Lee KT, et al. (2015) (9)	65	F	MCTD		Mostly asymptomatic	26	81	
Bishay RH, et al. (2016) (24)	76	F	SSc	Symptomatic anemia due to severe ulcerative esophagitis	None	76^a^	117^a^	
Dubourdieu V, et al. (2017) (23)	42	F	SSc		None	45^a^	108^a^	Earlobe
Drenthen LCA, et al. (2019) (22)	57	M	SSc	Aortic valve stenosis with intestinal angiodysplasia	None			Earlobe
Osman R, et al. (2021) (21)	52	F	SSc	Diarrhea	None			
Mertens J, et al. (2022) (20)	87	F	SSc		None			Earlobe
Amaral S, et al. (2022) (19)	47	F	SSc	Weight loss after sleeve gastrectomy	Lipothymia preceded by sweating, nausea, and dizziness	24	76	
Guzner A, et al. (2023) (18)	60	F	MCTD	Septic shock	None	<10	371	Earlobe
same as above	89	M	SSc	Septic shock, severe aortic stenosis	None	<60	>100	Earlobe
Sherman JJ, et al. (2025) (17)	81	F			None			
Our case	76	F	Dermatomyositis	Acute heart failure, sepsis	None	34	126	Earlobe

The glucose levels are listed in mg/dL.

M, male; F, female; SSc, systemic sclerosis; MCTD, mixed connective tissue disease.

a Values are read from line charts in the articles.

Interestingly, Guzner et al. reported two cases of artifactual hypoglycemia observed during recovery from septic shock in patients with scleroderma and Raynaud’s phenomenon (18). They considered that the vasoconstrictor endothelin-1 (ET-1), which is produced predominantly later in the course of sepsis, might have contributed to the pathogenesis. Artifactual hypoglycemia in our case also manifested during recovery from acute heart failure. ET-1, which is produced by endothelial cells, contributes to the pathogenesis of acute heart failure by inducing systemic and pulmonary vasoconstriction as well as promoting cardiac remodeling (26–28). Notably, ET-1 also plays a role in the pathogenesis of Raynaud’s phenomenon, and an ET-1 receptor antagonist (bosentan) has been used as a treatment option for this condition (14, 29, 30). Therefore, although the circulating level of ET-1 in our case was not available, which is one of the limitations of this case report, it is possible that hemodynamic changes associated with acute heart failure also contributed to the severity of Raynaud’s phenomenon and the emergence of artifactual hypoglycemia.

Several reports have suggested the co-occurrence of Raynaud’s phenomenon and myocardial infarction without coronary artery occlusion (31–33), prior to the widespread recognition of Takotsubo cardiomyopathy in Western countries. Although not all, at least some of these cases, such as the case of “reversible cardiogenic shock in an angry woman” with CREST syndrome (31), are consistent with Takotsubo cardiomyopathy. A retrospective cohort study demonstrated a significantly higher prevalence (16%) of Raynaud’s phenomenon in individuals with apical ballooning syndrome or Takotsubo cardiomyopathy than in those with ST-segment elevation myocardial infarction (0%) and control (2%) groups (34). Sympathetic nervous activation and high blood catecholamine levels as well as endothelial dysfunction, which is especially observed in secondary Raynaud’s phenomenon, are shared pathophysiologies of Takotsubo cardiomyopathy and Raynaud’s phenomenon (35, 36). The overlap in these mechanisms may explain the frequent co-occurrence of these two conditions.

Of note, after we switched to the earlobe-prick glucose measurement on day 21, an extremely low glucose value, which can be classified as level 2 or 3 (<54 mg/dL) hypoglycemia (37), was not observed; nonetheless, mild hypoglycemia classified as level 1 (<70 mg/dL) was still detected, especially before breakfast. As mentioned above, the patient developed a catheter-related bloodstream infection on day 19. We assume that the septic condition, in addition to the secondary adrenal suppression by taking oral prednisolone for more than 6 years, might be a possible explanation for this mild hypoglycemia. Although earlobe-prick glucose measurement noticeably attenuated the discrepancy against the actual venous glucose level in our case and in several previous reports (10, 18, 20, 22, 23), it should be noted that Raynaud’s phenomenon can also be observed in ear lobes, albeit less often than in the fingers (14, 38). Therefore, earlobe-prick and venous glucose levels must be compared before switching to earlobe-prick glucose monitoring in patients displaying artifactual hypoglycemia due to Raynaud’s phenomenon.

A clinical guideline from the Endocrine Society issued in 2009 recommended that the evaluation and management of hypoglycemia should be initiated only in patients with Whipple’s triad: a) symptoms and/or signs consistent with hypoglycemia, b) a low plasma glucose concentration (<55 mg/dL), and c) resolution of the symptoms after the plasma glucose concentration is raised (39). The patient in our case lacked symptoms associated with hypoglycemia. Clinicians should confirm if the patient satisfies Whipple’s triad before performing a thorough investigation to determine the cause of “hypoglycemia.” Considering the high frequency of steroid-induced diabetes and the concomitant necessity of glucose monitoring in patients with connective tissue diseases, clinicians should keep in mind that finger-stick glucose monitoring may display false-low values in patients with Raynaud’s phenomenon.

## Data Availability

The original contributions presented in the study are included in the article/supplementary material. Further inquiries can be directed to the corresponding author.
